# A Thymic Hyperplasia Case without Suppressing on Chemical Shift Magnetic Resonance Imaging

**DOI:** 10.1155/2018/7305619

**Published:** 2018-05-10

**Authors:** Tuan Phung, Thach Nguyen, Dung Tran, Nga Phan, Hung Nguyen

**Affiliations:** ^1^Radiology Department, Military Hospital 103, Vietnam Military Medical University, Hanoi, Vietnam; ^2^Anesthesiology Department, National Institute of Burns, Vietnam Military Medical University, Hanoi, Vietnam; ^3^Histopathology Department, Military Hospital 103, Vietnam Military Medical University, Hanoi, Vietnam; ^4^Neurology Department, Military Hospital 103, Vietnam Military Medical University, Hanoi, Vietnam

## Abstract

A 22-year-old woman with myasthenia gravis (MG) presented with ptosis and mild muscle weakness symptoms for one year. Computed tomography (CT) presented a diffuse bilobulate enlargement gland with a high density of soft tissue. Magnetic resonance imaging (MRI) showed the gland with no suppression on the opposed-phase chemical shift. After the thymic tumor diagnosis, she underwent thoracoscopic surgery for tumor resection. The postoperative histopathological finding was thymic lymphoid hyperplasia. This case suggests chemical shift MRI is not enough in distinguishing, and supplementary examination is essential to avoid unnecessary thymic biopsy and surgery.

## 1. Introduction

Thymic lymphoid hyperplasia (TLH) is very common in patients with myasthenia gravis. Contrary to true thymic hyperplasia, thymic lymphoid hyperplasia has diverse types and shapes. It can exhibit a normal shape and size, diffuse enlargement of both lobes, or a focal soft tissue mass [1]. Therefore, distinguishing thymic hyperplasia from thymic tumors is difficult. Inaoka et al.'s and Popa et al.'s studies showed that chemical shift magnetic resonance imaging is valuable in distinguishing thymic hyperplasia from thymic tumors [2, 3]. On the opposed-phase image, hyperplasia presents a signal intensity decrease, whereas the thymic tumor does not. We reported a thymic hyperplasia case that did not present the signal intensity decrease on chemical shift magnetic resonance imaging.

## 2. Case Report

A 22-year-old woman had ptosis and mild muscle weakness symptoms for one year. The symptoms were mild in the morning, severe in the evening, worse on exertion, and improved with rest. She had not diplopia, dyspnea, or dysphagia symptoms. Prostigmine and repetitive nerve stimulation tests were positive. She was diagnosed with myasthenia gravis and treated with corticosteroids and mytelase and her symptoms got better. After CT and MRI examination, she was diagnosed with a thymic tumor and underwent thoracoscopic surgery for tumor resection. The postoperative histopathological finding was thymic lymphoid hyperplasia.

CT examination was performed using a 2-section CT system (Siemens, Somatom Spirit, Germany) in a single-breath hold at end inspiration. Technical parameters included 120 kVp, 180 mAs, pitch of 1, section thickness of 5 mm, contiguous section interval, and 512 × 512 matrix without contrast agent intravenous injection. Observation was performed on soft tissue window W350, L100 HU.

MRI examination was obtained using a 1.5 T MRI unit (Intera Achieva, Philips Healthcare, Netherlands). She underwent transverse gradient-echo T1-weighted non-dual-echo in-phase and opposed-phase imaging, using an anterior-to-posterior phase-encoding direction, in separate breath holds. Imaging parameters included 350 mm field of view, 256 × 256 image matrix, 5 mm section thickness, 151 ms time repetition (TR), and in-phase and opposed-phase time echo (TE) of 4.6 and 2.3 ms. She also underwent axial T1-weighted and T2-weighted imaging without and with fat suppression and black blood technique with cardiac gate. Imaging parameters included TR 1000, 2000 ms, TE 10, 60 ms, and section thickness of 6 mm.

**Figure 1:**
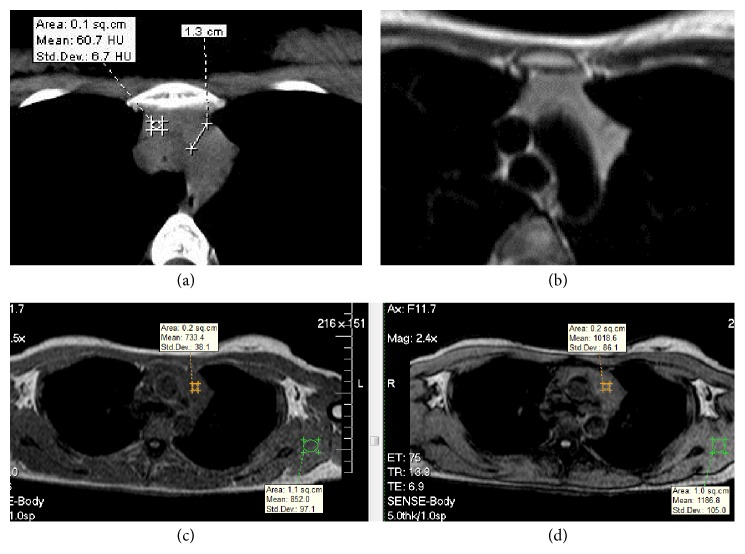
(a) CT image, (b) T2-weighted MRI, and (c) in-phase and (d) opposed-phase images presenting the gland without adipose tissue with CSR 1.0.

CT findings showed the relative homogeneous bilobulate gland with a high density of soft tissue (60.7 HU). The left lobe with a thickness of 13 mm was greater than the right lobe. The gland had straight margins. MRI findings presented the gland with an increased homogenous intensity on both T1-weighted and T2-weighted imaging. It was greater than in the wall muscle but lower than in the adipose tissue. Chemical shift magnetic resonance images demonstrated no decrease in signal intensity of the gland on the opposed-phase images relative to the in-phase images, suggesting the absence of fat component. The mean chemical shift ratio (CSR) was 1.0 (Figure 1).

**Figure 2:**
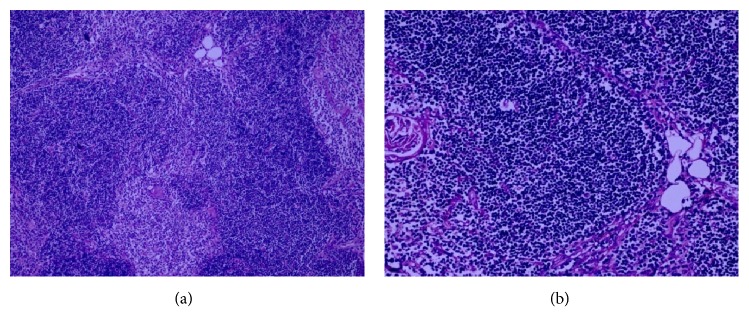
Microscopic images of thymic hyperplasia: great lymphoid follicles ((a) hematoxylin and eosin stain (H&E), magnification ×100), Hassall's corpuscles, and the rarity of adipose tissue ((b) hematoxylin and eosin stain (H&E), magnification ×200).

On gross examination, the resected thymus weighed 55 g, and the external surface was smooth and lobulated, triangle shaped, and yellowish, with a size of 25 × 20 × 10 mm. A microscopic examination revealed numerous reactive lymphoid follicles with large germinal centers in the thymic medulla. Lobules were separated by a thin fibrous septum and had a little fat tissue. In addition, there were an increased number of Hassall's corpuscles (Figure 2).

## 3. Discussion

Myasthenia gravis is an acquired autoimmune disease caused by an immune response in which IgG autoantibodies are produced against the acetylcholine receptors of the neuromuscular junction postsynaptic membrane. The thymic gland plays a very important role in the pathogenesis of MG. Above 90% of myasthenia gravis cases had abnormal thymus including 70% thymic lymphoid hyperplasia and 20% thymoma [4]. Differentiation between thymic lymphoid hyperplasia and thymoma is essential for surgical management. Thymectomy is strongly recommended in all thymoma cases. Conversely, the surgical indication for hyperplasia cases should be only considered with less effective conservation treatments [5]. CT is the most common diagnostic tool for distinguishing based on morphological assessment. On the CT, thymic hyperplasia manifests diffuse large gland image in two lobes while thymoma is in the form of localized soft tissue mass. However, Nicolaou' et al. study showed 45% hyperplasia cases with normal form, 35% two-lobe diffuse enlargement cases, and 20% soft tissue mass form cases [1]. Conversely, thymoma could show a diffuse enlargement gland. Therefore, chemical shift MRI is useful for distinguishing in atypical cases. It is able to detect microscopic fatty infiltration within the normal or hyperplastic thymus, which would be indistinct at CT, by showing homogeneous signal decrease on opposed-phase images relative to in-phase images, whereas signal loss is absent in thymoma that does not include fat [5]. However, fat infiltration in thymic gland occurs together with age. By evaluation of normal fatty replacement of the thymus, Inaoka et al. concluded that the CSR value should not be used in children under 16 years of age [6]. In another study, the author found that the CSR values of the tumor group and the hyperplasia group were 1.026 ± 0.039 and 0.614 ± 0.13, respectively [2]. Popa et al.'s study showed the tumor group CSR value and the hyperplasia group CSR value of 1.0398 ± 0.0244 and 0.4964 ± 0.1841, respectively [3]. These studies showed an accuracy of 100% for CSR, with no overlap in the range between the hyperplasia and tumor groups.

Besides CSR, another index also used to quantify fat tissue is signal intensity index (SII). The SII is often used when chemical shift imaging is obtained by a dual-echo technique. According to Priola et al., reference tissue was not only unnecessary but also incorrect because the tissue may contain a determined amount of fat [7]. The study of the author showed that SII had sensitivity (Se) and specificity (Sp) of 100% at cutoff point 8.92% and CSR had respective Se of 100% and Sp of 96.7% at cutoff point 0.849. No overlap was found for SII values between the two groups while CSR values overlapped in some cases [8]. However, the difference between two indexes was negligible. Furthermore, no other study has been published about the matter. In our study, due to using a non-dual-echo technique, we applied CSR to quantify fat tissue, which confirmed that this was a thymic hyperplasia case without fat.

Ackman et al. presented a pathologically proven case of normal thymus in a 21-year-old woman that demonstrated no fat replacement on the opposed-phase chemical shift MRI (CSR = 1.1) [9]. Priola et al. also reported a true hyperplasia case in a 60-year-old female being treated with corticosteroids without fat infiltration on chemical shift MRI (SII = −7.57%) [10]. Therefore, the soft tissue mass without fat in the position of thymus gland with the intermediate signal intensity on T1-weighted and T2-weighted MRI was not enough to determine the tumor, especially in young women. Seo et al.'s study about lipid-poor adrenal adenoma also showed the same results [11]. The author found that adrenal adenoma with the density on CT ≤ 20 HU (lipid-rich) had the sensitivity of adrenal-spleen ratio (ASR) on MRI of 100%, while adrenal adenoma with the density on CT > 30 HU (lipid-poor) had the sensitivity of ASR on MRI 61.5%.

With thymic hyperplasia cases without fat as our case, Priola's studies have suggested that diffusion-weighted MRI could be valuable because of its capability to reflect cell density and cellular architecture and to detect malignant tissues by demonstrating restricted diffusion and low (ADC) values. He reported two thymic hyperplasia cases without fat that was not detected on chemical shift imaging but was found on diffusion-weighted MRI due to the high ADC values 1.97 × 10^−3^ mm^2^/s and 2.47 × 10^−3^ mm^2^/s [5, 10]. His other study showed that diffusion-weighted MRI could be applied to distinguish thymic tumors from nonthymic tumors at cutoff ADC 1.625 × 10^−3^ mm^2^/sec with Se 96.8% and Sp 79.2% [12]. However, until now, this has been the only study using diffusion-weighted MRI to distinguish thymic tumors from normal and hyperplasia thymus. Studies of Razek et al. [13, 14], Usuda et al. [15], Seki et al. [16], and Gümüştaş et al. [17] always use diffusion-weighted MRI to distinguish benign from malignant tumors. Therefore, the matter should be further studied.

In thymic hyperplasia, histopathological characteristics revealed numerous reactive lymphoid follicles with prominent germinal centers in the thymic medulla. In adults, both normal thymus and thymic hyperplasia contain a great amount of fat tissue. However, in this case, postoperative histopathological findings showed only a few fat cells, which was not sufficient to detect the decrease of signal intensity on chemical shift MRI (Figure 2).

In conclusion, the normal and hyperplasia thymus glands present great fat infiltration. Conversely, thymoma does not show adipose tissue. Chemical shift magnetic resonance imaging is helpful in differentiating thymic lymphoid hyperplasia from thymic neoplasm. However, in a few cases, especially in young women, the chemical shift magnetic resonance imaging is not enough in distinguishing. Diffusion-weighted MRI and supplementary examination in these cases are essential to eliminate unnecessary thymic biopsy and thymectomy.
